# Muscle-Specific Ablation of Glucose Transporter 1 (GLUT1) Does Not Impair Basal or Overload-Stimulated Skeletal Muscle Glucose Uptake

**DOI:** 10.3390/biom12121734

**Published:** 2022-11-23

**Authors:** Shawna L. McMillin, Parker L. Evans, William M. Taylor, Luke A. Weyrauch, Tyler J. Sermersheim, Steven S. Welc, Monique R. Heitmeier, Richard C. Hresko, Paul W. Hruz, Francoise Koumanov, Geoffrey D. Holman, E. Dale Abel, Carol A. Witczak

**Affiliations:** 1 Departments of Kinesiology, Biochemistry & Molecular Biology, and Physiology, Brody School of Medicine, East Carolina Diabetes & Obesity Institute, East Carolina University, Greenville, NC 27858, USA; 2 Department of Anatomy, Cell Biology & Physiology, and Indiana Center for Musculoskeletal Health, Indiana University School of Medicine, Indianapolis, IN 46202, USA; 3 Indiana Center for Diabetes & Metabolic Diseases, Indiana University School of Medicine, Indianapolis, IN 46202, USA; 4 Departments of Pediatrics, and Cell Biology and Physiology, Washington University School of Medicine, St. Louis, MO 63130, USA; 5 Department of Health, University of Bath, Bath BA2 7AY, UK; 6 Department of Biology and Biochemistry, University of Bath, Bath BA2 7AY, UK; 7 Fraternal Order of Eagles Diabetes Research Center, Division of Endocrinology & Metabolism, Roy J. and Lucille A. Carver College of Medicine, University of Iowa, Iowa City, IA 52242, USA

**Keywords:** BAY-876, bio-LC-ATB-BGPA, glucose transport, GLUT1, mechanical overload, skeletal muscle, SLC2A1, synergist ablation

## Abstract

Glucose transporter 1 (GLUT1) is believed to solely mediate basal (insulin-independent) glucose uptake in skeletal muscle; yet recent work has demonstrated that mechanical overload, a model of resistance exercise training, increases muscle GLUT1 levels. The primary objective of this study was to determine if GLUT1 is necessary for basal or overload-stimulated muscle glucose uptake. Muscle-specific GLUT1 knockout (mGLUT1KO) mice were generated and examined for changes in body weight, body composition, metabolism, systemic glucose regulation, muscle glucose transporters, and muscle [^3^H]-2-deoxyglucose uptake ± the GLUT1 inhibitor BAY-876. [^3^H]-hexose uptake ± BAY-876 was also examined in HEK293 cells-expressing GLUT1-6 or GLUT10. mGLUT1KO mice exhibited no impairments in body weight, lean mass, whole body metabolism, glucose tolerance, basal or overload-stimulated muscle glucose uptake. There was no compensation by the insulin-responsive GLUT4. In mGLUT1KO mouse muscles, overload stimulated higher expression of mechanosensitive GLUT6, but not GLUT3 or GLUT10. In control and mGLUT1KO mouse muscles, 0.05 µM BAY-876 impaired overload-stimulated, but not basal glucose uptake. In the GLUT-HEK293 cells, BAY-876 inhibited glucose uptake via GLUT1, GLUT3, GLUT4, GLUT6, and GLUT10. Collectively, these findings demonstrate that GLUT1 does not mediate basal muscle glucose uptake and suggest that a novel glucose transport mechanism mediates overload-stimulated glucose uptake.

## 1. Introduction

Skeletal muscle is a key regulator of systemic glucose homeostasis due to its ability to take up glucose from the blood in both the fasted and post-prandial (insulin-stimulated) state [1,2]. Skeletal muscle glucose uptake is regulated by members of the facilitative glucose transporter family; with glucose transporter 1 (GLUT1) and glucose transporter 4 (GLUT4) being the two most abundant GLUT transporters detected in muscle [3]. Importantly, while numerous studies have demonstrated a critical role for GLUT4 in the regulation of insulin and contraction-stimulated muscle glucose uptake (see recent reviews [4,5,6,7]); the role of GLUT1 in the regulation of muscle glucose uptake is less clear.

The prevailing model for the regulation of skeletal muscle glucose uptake suggests that GLUT1 is responsible for controlling glucose uptake in the basal (non-stimulated) state (see recent reviews [5,6,8]). However, this model is largely based on studies demonstrating that transgenic mice with muscle-specific GLUT1 overexpression exhibit a 100–700% increase in basal muscle glucose uptake [9,10,11]; and to date no studies have demonstrated that a decrease in muscle GLUT1 expression or activity leads to a reduction in basal muscle glucose uptake. In addition, this current prevailing model conflicts with studies from global and muscle-specific GLUT4 knockout mice that have demonstrated a 30–90% decrease in basal muscle glucose uptake in the absence of changes in total muscle GLUT1 protein levels [12,13,14,15,16]. Thus, the role of endogenous GLUT1 in the regulation of basal muscle glucose uptake is presently unclear. Also in conflict with the current prevailing model for the role of GLUT1 in muscle are data suggesting that GLUT1 may be involved in the regulation of exercise-responsive muscle glucose uptake. These studies demonstrated a rapid and robust increase in skeletal muscle GLUT1 mRNA and protein levels in response to mechanical overload, a model of resistance exercise training [16,17]. These results are intriguing since recent work has demonstrated that 5 days of mechanical overload stimulates glucose uptake into skeletal muscle independent of GLUT4 [16]. Thus, collectively these findings suggest that GLUT1 may be the glucose transporter responsible for overload-stimulated muscle glucose uptake. Therefore, the objective of this study was to determine if muscle GLUT1 is necessary for either basal or mechanical overload-stimulated glucose uptake in skeletal muscle.

In this study, we generated the first described mouse model of muscle-specific GLUT1 ablation and examined whether GLUT1 expression in myocytes is required for the regulation of skeletal muscle glucose uptake in the basal, insulin-stimulated and mechanical overload-stimulated states. We further tested the requirement for GLUT1 in the regulation of basal and overload-stimulated muscle glucose uptake utilizing the chemical GLUT inhibitor, BAY-876. We examined muscles from the muscle-specific GLUT1 knockout mouse for evidence of possible compensation by other glucose transporters. Last, utilizing HEK293 cell lines that selectively express individual facilitative glucose transporters, we examined the specificity of BAY-876 to inhibit hexose uptake via glucose transporters, GLUT1-6 and GLUT10. Altogether our findings support the conclusion that GLUT1 does not mediate basal glucose uptake in skeletal muscle and suggest that a novel glucose transport mechanism mediates overload-stimulated muscle glucose uptake.

## 2. Materials and Methods

### 2.1. Mice

Procedures were performed in accordance with both the East Carolina University and the Indiana University School of Medicine Institutional Animal Care and Use Committees, as well as the NIH Guidelines for the Care and Use of Laboratory Animals.

Wild-type C57BL/6J (~6 wks. old; stock#000664) mice and muscle creatine kinase promoter-driven Cre recombinase (MCK-Cre; B6.FVB(129S4)-Tg(Ckmm-cre)5Khn/J; C57BL/6J strain; stock#006475) mice were obtained from The Jackson Laboratory [18]. GLUT1 LoxP mice (Slc2a1^tm1.1Stma^/AbelJ, C57BL/6J strain) were obtained from Dr. E. Dale Abel (University of Iowa) [19]. GLUT1 LoxP and MCK-Cre mice were bred to generate wild-type, GLUT1 LoxP, MCK-Cre, and muscle-specific GLUT1 knockout (mGLUT1KO) mice. Weaned mice were housed 2–4 mice/cage in static cages containing environmental enrichment in a specific pathogen free facility. Housing rooms were maintained at 21–22 °C with a 12 hr light/dark cycle, and mice assessed daily. A chow diet (Lab Diet Prolab^®^RMH3000 or Tekland Global 2018X, St. Louis, MO, USA) and water were provided ad libitum.

DNA isolated from tail snips was utilized to determine genotype using a KAPA mouse genotyping kit (Roche Diagnostics, cat#KK7352) and primers for GLUT1 (2.85F: 5′-CTGTGA GTTCCTGAGACCCTG-3′ and 2.9R: 5′-CCCAGGCAAGGAAG TAGTTC-3′) and MCK-Cre (CreF: 5′-TAAGTCTGAACCCGGTCTGC-3′ and CreR: 5′-GTGAAACAGCATTGCTGTCACTT-3′). Genotypes were confirmed using DNA isolated from skeletal muscle at the time of euthanasia and primers designed to assess GLUT1 gene recombination (2.85F described above and RecR: 5′-CCATGTGTGGAAGGC CATA-3′).

Mice were randomly assigned to an experimental group. For this study, sex and age-matched wild-type, MCK-Cre and GLUT1 LoxP mice (11–12 weeks old) were initially examined separately to assess possible alterations in body weight, body composition, muscle weight, whole-body metabolism, physical activity, fasting blood glucose and insulin levels, and muscle glucose uptake amongst these genotypes. Since there were no consistent differences in these measures amongst these genotypes for either sex (Supplemental Figure S1), data from the wild-type, MCK-Cre and GLUT1 LoxP mice was pooled into a single control (CON) group.

### 2.2. RT-qPCR

Tibialis anterior, extensor digitorum longus, soleus, and plantaris muscles were isolated, and placed in RNAprotect reagent (Qiagen, cat#76104, Hilden, Germany). Muscles (≤30 mg) were homogenized using a Bullet Blender tissue homogenizer (Next Advance, Troy, NY, USA), and total RNA isolated using the RNeasy fibrous tissue kit including DNase I treatment (Qiagen, cat#74704). RNA integrity was confirmed by gel electrophoresis-based visualization of rRNA using SYBR^®^ Safe stain. RNA levels were quantified using a NanoDrop One-C (ThermoScientific, Waltham, MA, USA), with sample yields of 417–544 ng RNA/mg for tibialis anterior muscles, 331–606 ng RNA/mg for extensor digitorum longus muscles, 607–828 ng RNA/mg for soleus muscles, 464–791 ng RNA/mg for sham-operated plantaris muscles, and 1132–2893 ng RNA/mg for overload-stimulated muscles. Sample purity was shown by A260/A280 ratios of 2.08–2.11. RNA was reverse-transcribed to cDNA (1 µg/20 µL reaction) using the iScript cDNA synthesis kit (Bio-Rad Laboratories, cat#1708891, Hercules, CA, USA), and cDNA stored at −80 °C.

RT-qPCR analyses were performed using methods standardized according to the Minimum Information for Publication of Quantitative Real-Time PCR Experiments (MIQE) guidelines [20]. RT-qPCR was performed using cDNA diluted (1:4) in nuclease-free water, iTaq Universal SYBR green master mix (Bio-Rad Laboratories, cat#1725121), and 400 nM of the gene-specific primers described in Table 1. Individual reactions (10 µL) were carried out using the iTaq Universal SYBR green master mix standard PCR cycling conditions in a CFX384 Touch Real-Time PCR detection system with CFX Maestro v1.1 software (Bio-Rad Laboratories).

Specificity of PCR products was confirmed by melt curves. Primer PCR efficiency was calculated from calibration curves [parameters (ranges): slope (−3.57 to −3.30), y-intercept (25.6–40.5), and r^2^ values (0.99–1.00)] and verified to be linear over a ≥100-fold range for all primers (Table 1). GLUT gene expression was calculated using the geometric mean of 3 reference genes [i.e., hydroxyacyl glutathione hydrolase (*HAGH*), ribosomal protein S17 (*RPS17*), and signal recognition particle 14 (*SRP14*)], and the ΔΔCt method. The reference genes were selected based on a lack of change in response to genotype or treatment group.

### 2.3. Body Weight & Composition

Mice were weighed to ±0.1 g. Lean and fat mass was assessed using an EchoMRI™-700 body composition analyzer.

### 2.4. Metabolic Cages

Mice were individually housed in Pheno/LabMaster metabolic cages (TSE Systems, Chesterfield, MO, USA) at 21–22 °C with a 12 h light/dark cycle for 2 days for measures of the volume of oxygen consumption (VO_2_), volume of carbon dioxide production (VCO_2_), energy expenditure, and spontaneous physical activity.

### 2.5. Glucose Tolerance Test

Mice were fasted 12–14 h overnight. Blood was sampled from the tail to measure glucose levels with a glucometer (Onetouch^®^Ultra^®^2, Milpitas, CA, USA) and insulin levels by ELISA (Crystal Chem Inc, cat#90080, Elk Grove Village, IL, USA). Glucose was injected (2 g/kg lean mass, intraperitoneal) and blood glucose levels measured 15, 30, 60 and 120 min later.

### 2.6. Unilateral Synergist Muscle Ablation Surgery

Mice were anesthetized with isoflurane gas (2–4%) and injected with the analgesic buprenorphine (0.05 mg/kg, subcutaneous). Plantaris muscle overload was induced via the unilateral surgical ablation of the distal two-thirds of the gastrocnemius and soleus using methods described by our lab [16,21,22]. The contralateral leg was sham operated as control. Mice received buprenorphine for 3 days and were monitored every day. After 5 days, mice were anesthetized with pentobarbital sodium (80–100 mg/kg, intraperitoneal) and euthanized by cervical dislocation. Muscles were isolated and weighed to ±0.1 mg.

### 2.7. Muscle Glucose Uptake

In vivo muscle [^3^H]-2-deoxyglucose uptake was assessed as described by our group [23]. Mice were fasted 12–14 h overnight and anesthetized with pentobarbital sodium (80–100 mg/kg, intraperitoneal). Blood was collected from the tail to assess basal glucose and radioactivity, and [^3^H]-2-deoxyglucose (0.33 µCi/g mouse body weight) dissolved in saline injected retro-orbitally. Blood was sampled every 5–10 min for 45 min to assess glucose and [^3^H]-2-deoxyglucose levels. Mice were euthanized by cervical dislocation and muscles frozen in liquid nitrogen. Muscles were solubilized, aliquots treated with 7% perchloric acid or 0.3 M Ba(OH)_2_/ZnSO_4_, and samples removed for liquid scintillation counting of the [^3^H]-label. Glucose uptake rates were calculated as previously described [24].

Ex vivo muscle [^3^H]-2-deoxyglucose uptake was assessed as described by our group [16,22,23]. Mice were fasted 12–14 h overnight, anesthetized with pentobarbital sodium (80–100 mg/kg, intraperitoneal) and euthanized by cervical dislocation. Isolated muscles were pre-incubated in 95% O_2_-5% CO_2_-gassed, 37 ºC Krebs-Ringer-Bicarbonate buffer [(KRBB): 117 mM NaCl, 4.7 mM KCl, 2.5 mM CaCl_2_, 1.2 mM KH_2_PO_4_, 1.2 mM MgSO_4_, 24.6 mM NaHCO_3_] containing 2 mM sodium pyruvate. Muscles were stimulated with a maximal dose of insulin (50 mU/mL; Sigma-Aldrich, cat#11376497001, St. Louis, MO, USA), treated with the chemical GLUT inhibitor, BAY-876 (0.05 µM, 1 µM and 5 µM; Sigma-Aldrich, cat#SML1774), or treated with the vehicle control, DMSO (0.2%). Muscles were incubated in 30 ºC KRBB containing 1.5 µCi/mL [^3^H]-2-deoxy-D-glucose, 0.45 µCi/mL [^14^C]-mannitol, 1 mM 2-deoxy-D-glucose, and 7 mM mannitol for 10 min, and frozen in liquid nitrogen. Muscles were solubilized in 1M NaOH at 80 °C and neutralized with 1 M HCl. Samples were centrifuged at 10,000× *g* for 1 min. Aliquots were removed for liquid scintillation counting of the [^3^H]- and [^14^C]-labels, and glucose uptake rates calculated.

### 2.8. Muscle Glycogen

Glycogen content was assessed as previously described by our lab in solubilized muscle fractions from the ex vivo muscle glucose uptake experiments using a hexokinase reagent (Eagle Diagnostics, cat#2820, Cedar Hill, TX, USA) as previously described by our lab [22].

### 2.9. Muscle Cell Surface GLUT Capture

Cell surface GLUT4 protein levels were detected with the membrane impermeable biotinylated bis-glucose photoaffinity label, 4,4-O-[2-[2-[2-[2-[2-[6-(biotinylamino) hex-anoyl]-amino]ethoxy]ethoxy]ethoxy]-4-(1-azi-2,2,2,-trifluoroethyl) benzoyl]amino-1,3-propanediyl]-bis-D-glucose (bio-LC-ATB-BGPA) [25], using methods adapted from Ryder et al. [26]. Muscles were incubated in bio-LC-ATB-BGPA (400 µM) at 18 °C for 8 min, UV-irradiated (300 nm, 2 × 3 min) in a Rayonet Chamber Reactor RMR-600, and frozen in liquid nitrogen. Muscles were homogenized in lysis buffer [20 mM HEPES (pH 7.2), 1 mM EDTA, 225 mM sucrose, 10 mM Na_4_P_2_O_7_, 100 mM NaF, 2 mM NaVO_4_, 3 mM benzamidine, 1 mM phenyl-methyl-sulfonyl-fluoride, 10 μg/mL aprotinin and 10 μg/mL leupeptin], and centrifuged at 277,000× *g* for 50 min. Pellets were resuspended in buffer [20 mM PBS (pH 7.2), 1 mM EDTA, 1% IGEPAL, 10 mM Na_4_P_2_O_7_, 100 mM NaF, 2 mM NaVO_4_, 3 mM benzamidine, 1 mM phenylmethylsulfonyl-fluoride, 10 µg/mL aprotinin and 10 µg/mL leupeptin], rotated at 4 °C for 1 h, and centrifuged at 13,000× *g* for 30 min. Equal protein amounts of supernatants were affinity precipitated using streptavidin-agarose beads (ThermoScientific, cat#20347) at 4 °C overnight. Beads were washed, and proteins eluted by heating at 95 °C for 20 min in Laemmli’s buffer. Dithiothreitol (20 mM) was added, and samples subjected to immunoblot analyses as described below.

### 2.10. Immunoblot Analyses

GLUT4 immunoblot analyses were performed using standard methods described by our lab [16,22]. Plantaris muscles were homogenized in lysis buffer [20 mM Tris-HCl pH 7.4, 5 mM EDTA, 10 mM Na_4_P_2_O_7_, 100 mM NaF, 2 mM Na_3_VO_4_, 1% IGEPAL^®^ CA-630, 1.5 µM aprotinin, 3 mM benzamidine, 23.4 µM leupeptin and 1 mM phenylmethyl- sulfonyl fluoride] using a Bullet Blender tissue homogenizer (Next Advance). Samples were rotated end-over-end at 4 °C for 1 h, centrifuged at 14,000× *g* for 30 min, and lysate protein concentrations determined.

Lysates or bio-LC-ATB-BGPA affinity precipitates were subjected to SDS-PAGE using 10% acrylamide gels and proteins transferred onto 0.2 µm nitrocellulose membranes. Membranes were blocked with a 5% bovine serum albumin, 1× Tris-buffered-saline +0.1% Tween (5% BSA + 1×TBST) solution for 1 hr at 20–22 °C. Membranes were incubated with GLUT4 antibody (Millipore, cat#07-1404, lot#2890837, RRID# AB_1587080; 1:2000 in 5% BSA+1×TBST solution, Burlington, MA, USA) at 4 °C overnight. The GLUT4 antibody was previously validated using muscle-specific GLUT4 knockout muscles [16]. Membranes were incubated with rabbit-horseradish peroxidase secondary antibody (Cell Signaling Technology, cat#7074, lot#30, RRID#AB_2099233; 1:5000 in 5% BSA + 1×TBST solution, Danvers, MA, USA) for 1 h at 20–22 °C. Membranes were incubated with chemiluminescent substrate (Perkin Elmer, cat#NEL105001EA, Waltham, MA, USA) and luminescence visualized using a Bio-Rad Chemidoc imager. Densitometric analyses were performed using Bio-Rad Image Lab™ software.

### 2.11. GLUT-HEK293 Cell Hexose Uptake

[^3^H]-hexose uptake into HEK293 cells selectively expressing human GLUT1-6 or GLUT10 was assessed as previously described [27]. Endogenous GLUT1 expression was knocked down in all cell lines except for the GLUT1 line, and levels of human GLUT1-6 and GLUT10 transcripts measured by RT-qPCR as has been previously reported [28], and shown in Table 2. Cells were plated at 4 × 10^5^ cells/well in 24-well plates pre-treated with polyethyleneimine (25 µg/mL) and maintained at 37 °C overnight. Cells were incubated in glucose-free Hank’s Balanced Salt Solution [HBSS: 146 mM NaCl, 4.7 mM KCl, 0.6 mM MgSO_4_, 1.6 mM NaHCO_3_, 0.13 mM NaH_2_PO_4_, 2.5 mM CaCl_2_, 20 mM HEPES (pH 7.3)] at 37 °C for 30 min containing DMSO or BAY-876 as indicated in the figure legends. Cells were incubated in HBSS containing [^3^H]-2-deoxyglucose (50 µM, 1 μCi/mL) for 4 min or [^3^H]-D-fructose (4 µM, 1 μCi/mL) for 2 min. Cells were washed in ice-cold 1× PBS and lysed with 1× PBS + 0.1% Triton X-100. Samples were removed for liquid scintillation counting of the [^3^H]-label, and hexose uptake calculated.

### 2.12. Statistical Analysis

Data sets were screened once for outliers defined as data points whose value exceeded the mean ± 2× standard deviation for that group. Any identified outliers were removed prior to statistical analyses. Statistical analyses were performed using GraphPad Prism software (GraphPad Software, San Diego, CA, USA) and significance defined as *p* < 0.05. The data are reported as mean ± standard deviation, except for the glucose uptake data from the GLUT-HEK293 cells which is reported as mean ± standard error. The statistical test and sample size for each data set are indicated in the figure legends.

## 3. Results

### 3.1. mGLUT1KO Mice Are Viable with No Alterations in Body Size, Body Composition, Whole Body Metabolism or Systemic Glucose Regulation

**Figure 1:**
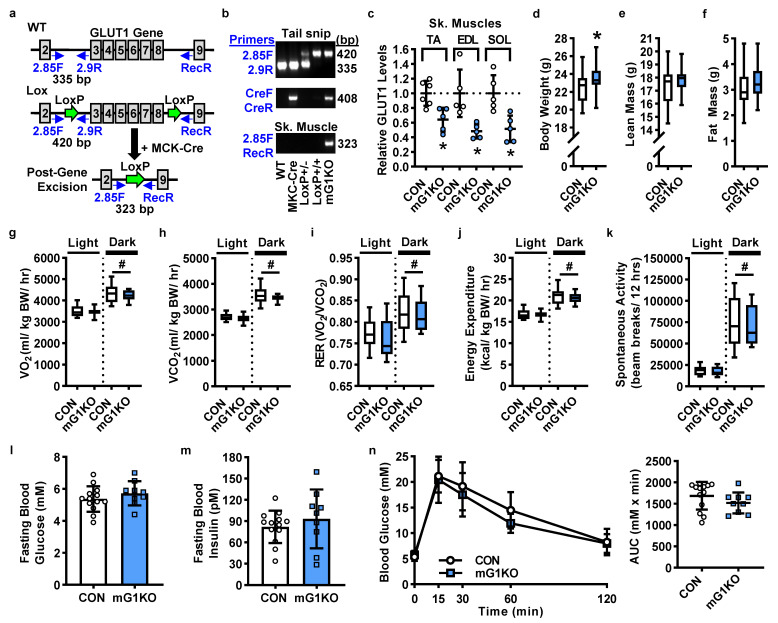
Muscle-specific GLUT1 knockout (mG1KO) mice are viable but do not exhibit impairments in body weight, body composition, whole body metabolism, spontaneous physical activity, or systemic glucose regulation. (**a**) Schematic representation of the GLUT1 gene including the location of the LoxP sites and the primers used for mouse genotyping and post-gene recombination. (**b**) Representative images of the mouse genotyping PCR reaction products using DNA isolated from tail snips (top 2 images). The excision of GLUT1 exons 3–8 was confirmed in skeletal muscle samples obtained from wild-type (WT), muscle creatine kinase promoter-driven Cre recombinase (MCK-Cre) transgenic, GLUT1 LoxP+/−, GLUT1 LoxP+/+, and mG1KO mice post-euthanasia (bottom image). (**c**) GLUT1 mRNA levels assessed in tibialis anterior (TA), extensor digitorum longus (EDL), and soleus (SOL) muscles by RT-qPCR. *N* = 5–6 muscles/group. (**d**–**f**) In female control (CON) and mG1KO mice, the following were assessed: (**d**) body weight; (**e**) lean mass; and (**f**) fat mass. *N* = 22–34 mice/group. (**g**–**k**) Female mice were individually housed in metabolic cages at 21–22 °C for indirect calorimetry measures in both the light and dark cycles. Measures included: (**g**) oxygen consumption (VO_2_); (**h**) carbon dioxide production (VCO_2_); (**i**) respiratory exchange ratio (RER); and (**j**) energy expenditure. (**k**) Infrared sensors detected spontaneous physical activity. *N* = 14–24 mice/group. (**l**–**n**) Female mice were fasted overnight. Blood was collected from the tail to assess: (**l**) glucose and (**m**) insulin levels. (**n**) Mice received an intraperitoneal injection of glucose (2 g/kg lean mass), and blood glucose measured 15, 30, 60 and 120 min later. The area under the curve was calculated (inset). *N* = 9–13 mice/group. Data are presented as individual data points with the mean ± standard deviation (**c**,**l**–**n** inset); box and whisker plots with error bars denoting min and max (**d**–**k**); and mean ± standard deviation (**n**). Statistical significance was defined as *p* < 0.05, determined using *t*-tests (**c**–**f** and **l**–**n**) or repeated measures two-way ANOVA with Sidak’s posthoc analysis (**g**–**k**), and denoted by ‘*’ vs. control (CON) mice or “#” vs. main effect (light vs. dark cycle).

A muscle specific-GLUT1 knockout (mGLUT1KO) mouse was generated by breeding two established mouse models: a muscle creatine kinase promoter-driven Cre recombinase (MCK-Cre) mouse [12,13,16,18] with a GLUT1 LoxP mouse [19,29,30,31,32,33] (Figure 1a). Genotypes were initially determined by PCR using tail snip DNA (Figure 1b, top and middle image). Genotypes were confirmed using DNA obtained from skeletal muscle biopsies acquired at euthanasia, verifying the removal of exons 3-8 in the GLUT1 gene in the muscle from the mGLUT1KO mice (Figure 1b, bottom image). A 35–50% reduction in GLUT1 mRNA levels was observed in multiple muscles from the mGLUT1KO mice (Figure 1c). This magnitude of reduction in GLUT1 gene expression is consistent with the heterogeneous cell type composition of whole muscle tissue and the known presence of GLUT1 in many cell types such as endothelial cells and neurons [34,35,36,37,38]. Unlike global GLUT1 deletion which results in embryonic lethality [39], mGLUT1KO mice were viable and did not exhibit decreases in body weight or body composition (Figure 1d–f, female mice; Supplementary Figure S2a–c, male mice).

To determine whether mGLUT1KO mice exhibit alterations in whole body metabolism, mice were placed in metabolic cages. mGLUT1KO mice did not exhibit changes in VO_2_, VCO_2_, respiratory exchange ratio, energy expenditure, or spontaneous physical activity in either the light or dark cycles (Figure 1g–k, female mice; Supplementary Figure S2d–h, male mice).

To determine whether mGLUT1KO mice exhibit alterations in systemic glucose regulation, mice were fasted overnight and glucose tolerance tests performed. mGLUT1KO mice did not exhibit impairments in fasting blood glucose levels, fasting blood insulin levels, or glucose tolerance (Figure 1l–n, female mice; Supplementary Figure S2i–k, male mice).

### 3.2. GLUT1 Expression in Muscle Is Not Required for Basal, Insulin-Stimulated or Overload-Stimulated Glucose Uptake

**Figure 2:**
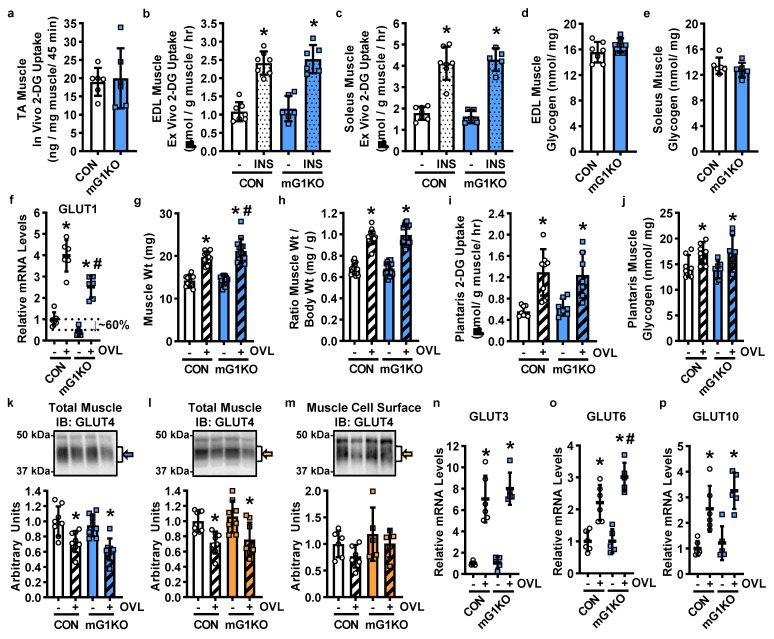
Effect of muscle-specific GLUT1 knockout (mG1KO) on skeletal muscle glucose uptake, glycogen content, and glucose transporter levels. (**a**) Female control (CON) and mG1KO mice were fasted overnight and anesthetized with pentobarbital sodium. Mice were injected with [^3^H]-2-deoxyglucose (2-DG) and in vivo glucose uptake assessed in tibialis anterior (TA) muscles. *N* = 6 muscles/group. (**b**,**c**) Ex vivo glucose uptake was assessed in the absence (-) or presence of insulin (INS; 50 mU/mL) in the (**b**) extensor digitorum longus (EDL), and (**c**) soleus muscles. *N* = 6–7 muscles/group. (**d**,**e**) Glycogen content was assessed in (**d**) EDL, and (**e**) soleus muscles. *N* = 6–7 muscles/group. (**f**–**o**) Mice underwent unilateral synergist muscle ablation surgery to induce plantaris muscle overload (OVL). After 5 days, plantaris muscles were excised. (**f**) Muscles from female mice were processed to assess GLUT1 mRNA levels by RT-qPCR. *N* = 6 muscles/group. (**g**,**h**) Muscles from female mice were weighed to ±0.1 mg. The ratio of muscle weight to body weight was calculated. *N* = 12–14 muscles/group. (**i**) Muscles from female mice were incubated in [^3^H]-2-DG and glucose uptake rates calculated. *N* = 7–8 muscles/group. (**j**) Glycogen content was assessed in plantaris muscles from female mice. *N* = 7 muscles/group. (**k**,**l**) Muscles were processed to assess total muscle GLUT4 protein levels by immunoblot analysis. Representative images and data provided for (**k**) female and (**l**) male mice. *N* = 8 muscles/group. (**m**) Muscles from male mice were incubated with the membrane impermeant biotinylated bis-glucose photolabeling reagent, bio-LC-ATB-BGPA, and processed to assess cell surface GLUT4 protein levels by immunoblot analysis. Representative images and data provided. *N* = 5–6 muscles/group. (**n**–**p**) Muscles from female mice were processed to assess GLUT3, GLUT6 and GLUT10 mRNA levels by RT-qPCR. *N* = 6 muscles/group. Data are presented as individual data points with the mean ± standard deviation. Statistical significance was defined as *p* < 0.05, assessed by repeated measures two-way ANOVA with Sidak’s posthoc analysis, and denoted by ‘*’ vs. sham-operated muscles (-) and “#” vs. control (CON) mice.

To determine whether mGLUT1KO mice exhibit impairments in basal or insulin-stimulated glucose uptake, [^3^H]-2-deoxyglucose was examined in tibialis anterior muscles in vivo as well as in extensor digitorum longus and soleus muscles ex vivo. None of the muscles from the mGLUT1KO mice exhibited alterations in basal or insulin-stimulated glucose uptake (Figure 2a–c, female mice; Supplementary Figure S2l–n, male mice). This lack of a change in glucose uptake was not due to alterations in muscle glycogen levels (Figure 2d,e, female mice; Supplementary Figure S2o,p, male mice).

To determine whether mGLUT1KO mice have impaired overload-stimulated muscle growth or glucose uptake, mechanical overload was induced in plantaris muscles via unilateral synergist muscle ablation. After 5 days, plantaris muscles were examined for changes in GLUT1 expression, hypertrophic growth, and glucose uptake rates. An ~60% reduction in GLUT1 mRNA levels was observed in non-stimulated plantaris muscles, and a ~40% reduction in GLUT1 mRNA levels was observed in overload-stimulated muscles from the mGLUT1KO mice (Figure 2f, female mice). Overload-stimulated muscle hypertrophy was not impaired in the mGLUT1KO mice (Figure 2g, female mice; Supplementary Figure S1q, male mice). Overload-stimulated glucose uptake ~130% in muscles from the control mice, and this effect was not impaired in the mGLUT1KO mice (Figure 2h, female mice; Supplementary Figure S2r, male mice). This lack of a change in glucose uptake was not due to alterations in muscle glycogen levels (Figure 2i, female mice; Supplementary Figure S2s, male mice).

To assess possible compensation by GLUT4, total muscle GLUT4 levels were assessed by immunoblot analyses. Overload decreased total GLUT4 protein levels in muscles from control and mGLUT1KO mice (Figure 2j, female mice; Figure 2k, male mice). To assess cell surface GLUT4 protein levels, muscles were incubated in the membrane impermeable biotinylated bis-glucose photoaffinity label, bio-LC-ATB-BGPA, and translocated GLUT4 assessed by immunoblot analyses. Cell surface GLUT4 levels were not increased in muscles from the mGLUT1KO mice (Figure 2l, male mice).

Previous work in mouse muscle demonstrated that mechanical overload increases the expression of GLUT3, GLUT6, and GLUT10 [16,17]. To determine whether the levels of these mechanosensitive GLUTs were elevated in the mGLUT1KO mice, RT-qPCR was performed. Overload stimulated an increase in the expression of GLUT3, GLUT6 and GLUT10 in muscles from both the control and mGLUT1KO mice (Figure 2m–o, female mice); but only GLUT6 levels were higher in muscles from the mGLUT1KO mice compared to controls.

### 3.3. BAY-876 Inhibits Overload-Stimulated but Not Basal Muscle Glucose Uptake

**Figure 3:**
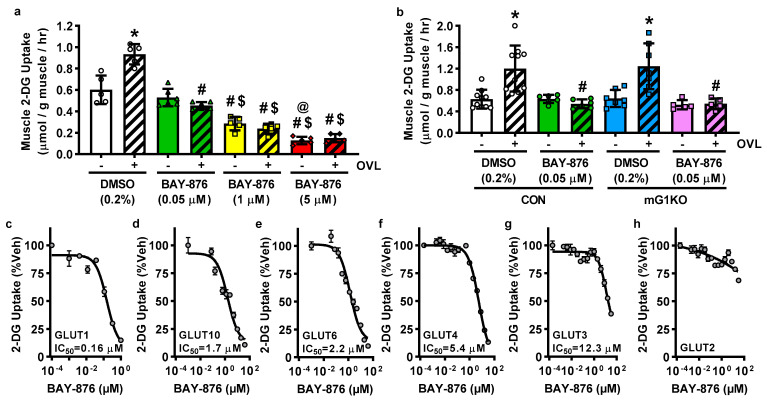
Effects of the chemical GLUT inhibitor, BAY-876, on glucose uptake in skeletal muscle and in glucose transporter (GLUT)-expressing HEK293 cells. (**a**,**b**) Female wild-type mice, or female control (CON) and muscle-specific GLUT1 knockout (mG1KO) mice, underwent unilateral synergist muscle ablation surgery to induce plantaris muscle overload (OVL). After 5 days, mice were anesthetized and the plantaris muscles excised. (**a**) Muscles from wild-type mice were incubated in the presence of BAY-876 (0.05 µM, 1 µM or 5 µM) or DMSO (0.2%) for 60 min, incubated in [^3^H]-2-deoxyglucose (2-DG), and glucose uptake rates calculated. *N* = 5–6 muscles/group. (**b**) Muscles from CON and mG1KO mice were incubated in the presence of BAY-876 (0.05 µM) or DMSO (0.2%) for 60 min, incubated in [^3^H]-2-DG, and glucose uptake rates calculated. *N* = 5–10 muscles/group. (**c**–**h**) HEK293 cells that stably express human GLUT transporters were incubated with vehicle (Veh; 0.45% DMSO) or BAY-876 for 30 min, and [^3^H]-2-DG uptake assessed. Data was collected for each of the following GLUT transporters: (**c**) GLUT1; (**d**) GLUT10; (**e**) GLUT6; (**f**) GLUT4; (**g**) GLUT3; and (**h**) GLUT2. Data are presented as individual data points with the mean ± standard deviation (**a**,**b**). *N* = 3 independent experiments, *N* = 3 replicates per experiment. Data are presented as the percent of hexose uptake relative to vehicle treated cells ± standard error, and the IC_50_ values determined using least squares non-linear regression analyses (**c**–**h**). Statistical significance was defined as *p* < 0.05, determined by repeated measures two-Way ANOVA with Sidak’s posthoc analysis, and denoted by ‘*’ vs. sham-operated controls (-), ‘#’ vs. DMSO, ‘$’ vs. 0.05 µM BAY-876, and ‘@’ vs. 1 µM BAY-876.

To assess possible functional compensation by GLUT3 in the muscles from the mGLUT1KO mice, muscles were incubated in varying doses of the recently developed chemical GLUT1-4 inhibitor, BAY-876 [40], and glucose uptake assessed. A low dose of BAY-876 (0.05 μM) completely impaired overload-stimulated, but not basal muscle glucose uptake (Figure 3a, female mice). Increasing BAY-876 to 1 μM or 5 μM impaired both basal and overload-stimulated muscle glucose uptake. To assess the specificity of low dose BAY-876, muscles from mGLUT1KO mice were incubated with BAY-876 and [^3^H]-2-deoxyglucose uptake assessed. BAY-876 (0.05 μM) impaired overload-stimulated but not basal glucose uptake in muscles from both control and mGLUT1KO mice (Figure 3b, female mice).

### 3.4. BAY-876 Inhibits Glucose Uptake via Multiple Facilitative Glucose Transporters

The initial work describing the inhibitory capabilities of BAY-876 on GLUT1, GLUT2, GLUT3 and GLUT4 used cell viability and not hexose transport assays. To assess the ability of BAY-876 to inhibit hexose uptake via facilitative glucose transporters, [^3^H]-hexose uptake was examined in HEK293 cell lines with stable knockdown of GLUT1 and selective expression of individual human GLUT1-6 and GLUT10 [27]. As shown in Figure 3c–h, BAY-876 impaired [^3^H]-2-deoxyglucose uptake through GLUT1 (IC_50_ = 0.16 μM), GLUT10 (IC_50_ = 1.7 μM), GLUT6 (IC_50_ = 2.2 μM), GLUT4 (IC_50_ = 5.4 μM) and GLUT3 (IC_50_ = 12.3 μM), but not GLUT2. Fructose transport through GLUT5 was not impaired by BAY-876 (Supplementary Figure S3).

## 4. Discussion

The data presented in this study argues against the prevailing model that the role of GLUT1 in skeletal muscle is to facilitate glucose uptake in the basal (non-stimulated) state. The findings show that neither the muscle-specific loss of GLUT1 expression nor the chemical inhibition of GLUT1 activity prevented basal muscle glucose uptake. In addition, the findings show that despite an increase in muscle GLUT1 expression in response to mechanical overload, GLUT1 is not required for overload-stimulated skeletal muscle glucose uptake.

Previous work in muscle-specific GLUT1 overexpression mice demonstrated that a 100–700% increase in basal muscle glucose uptake corresponds with a 40–50% decrease in fasting blood glucose levels and a robust enhancement in glucose tolerance compared to controls [9,10,11,41]. Collectively, these findings were interpreted to indicate that the role of GLUT1 in skeletal muscle is to regulate glucose uptake in the basal state. However, findings from this study demonstrate that the loss of GLUT1 expression in muscle was not sufficient to impair basal glucose uptake in mouse tibialis anterior in vivo, or in extensor digitorum longus, soleus or plantaris muscles ex vivo. Consistent with this lack of a decrease in basal muscle glucose uptake, the loss of GLUT1 expression in muscle had no effect on fasting blood glucose levels or glucose tolerance. Although these findings argue against a role for GLUT1 in regulating basal glucose uptake in muscle, they are consistent with findings from global and muscle-specific GLUT4 knockout mice that demonstrated significant reductions (30–90%) in basal muscle glucose uptake in the absence of changes in muscle GLUT1 levels [12,13,14,15,16]. It is unlikely that the absence of any observed change in systemic glucose homeostasis is due to a compensatory increase in the suppression of hepatic gluconeogenesis or an increase in fatty acid or *β*-hydroxybutyrate utilization since mGLUT1KO mice showed no increase in blood insulin levels, and no decrease in respiratory exchange ratio.

Both mechanical overload of skeletal muscle and pressure overload of cardiac muscle induce GLUT1 expression [29]. However, strikingly while cardiomyocyte-specific GLUT1KO mice have impaired pressure overload-stimulated glucose uptake and glucose oxidation [29], mGLUT1KO mice have no impairments in mechanical overload-stimulated muscle glucose uptake. The reason underlying this difference between striated muscles is presently unknown. However, it cannot be explained by a compensatory increase in GLUT4, since neither total nor cell surface GLUT4 levels were elevated in muscles from the mGLUT1KO mice. The lack of an impairment in mechanical overload-stimulated glucose uptake in the mGLUT1KO mice could instead suggest that either GLUT1 is part of a broad induction of glucose transport mechanisms that are redundant and functionally overlapping and/or that GLUT1 facilitates the transport of another molecule. In addition to D-glucose and 2-deoxy-D-glucose, GLUT1 has been shown to transport D-mannose [42], D-galactose [42], dehydroascorbic acid [43] and glucosamine [44]. Glucosamine has been implicated in the hexosamine pathway and O-linked N-acetylglucosamine (O-GlcNac) posttranslational modifications [45]; and O-GlcNac levels have recently been reported to increase in response to muscle overload [22]. Thus, future studies are needed to determine if a relationship exists linking changes in GLUT1 expression to changes in muscle O-GlcNAc protein modifications and muscle growth [45].

The reason underlying the simultaneous induction of GLUT1, GLUT3, GLUT6 and GLUT10 following mechanical overload in skeletal muscle is currently unknown. It is possible that each transporter could be induced to transport a discrete substrate that is utilized to fuel the energetic and biosynthetic demands of muscle hypertrophic growth. For example, GLUT1 could transport glucosamine for O-GlcNac synthesis [22,44,45]; GLUT3 could transport D-galactose for N-glycan galactosylated posttranslational modifications or conversion to galactonate for metabolism in the pentose phosphate pathway [46,47]; GLUT6 could transport D-glucose for ATP generation [48]; and GLUT10 could be transporting dehydroascorbic acid for redox buffering [49,50,51]. It is also possible that each transporter could be expressed in one or more distinct cell types found within overload-stimulated muscle tissue. For example, while GLUT1 has been detected in myocytes and endothelial cells [34,35,36,37,38]; GLUT3 has been detected in myocytes and neurons [52,53]; GLUT6 in myocytes, endothelial, and immune cells [54,55,56]; and GLUT10 in myocytes and vascular smooth muscle cells [53,57]. Prior work in rat muscle has demonstrated that 1 week of mechanical overload is sufficient to increase the capillary density to muscle fiber ratio [58]; providing a possible explanation for the induction of endothelial cell-derived GLUTs. Additional studies are needed to determine the preferred physiological substrate(s) and cell type expression of all of the mechanosensitive facilitative glucose transporters detected in both unstimulated and overload-stimulated skeletal muscles.

Three commercially available GLUT1 antibodies were evaluated for their ability to detect GLUT1 protein levels in the sham and overload-stimulated muscles from the control and mG1KO mice (Supplementary Figure S4). These antibodies had been used in published studies that contained tissue-specific GLUT1 knockdown or knockout tissue samples [19,32,33,59,60,61]. Muscle lysates were treated with and without the enzyme, PNGase F, to remove glycolysation on the GLUT1 proteins. While the GLUT1 immunoreactive band pattern was very similar amongst the three GLUT1 antibodies, numerous bands of varying molecular weights were detected. Given the lack of clarity on which band(s) truly represented GLUT1 in the muscle samples, the data were not quantified. Additional studies are needed with more specific antibodies to assess GLUT1 protein levels in the muscles from the mG1KO mice.

The identity of the GLUT transporter responsible for mechanical overload-stimulated muscle glucose uptake is still unknown. However, this study added an important new piece of information regarding its identity, i.e., it is inhibited by BAY-876 at ≤0.05 μM. This result was initially surprising since in the GLUT-expressing HEK293 cells, [^3^H]-2-deoxyglucose uptake was minimally impaired via GLUT1 (inhibited by 23.1%), GLUT3 (inhibited by 5.9%), GLUT4 (inhibited by 1.9%), GLUT6 (inhibited by 1.8%), and GLUT10 (inhibited by 9.7%) at 0.05 µM BAY-876. While these data could suggest that GLUT3, GLUT6 and GLUT10 can be excluded as possible contributors to overload-stimulated muscle glucose uptake, this interpretation should be considered with caution as the possibility remains that BAY-876 could impact endogenous GLUTs in skeletal muscle with kinetics that differ from the findings obtained in the GLUT1-6 and GLUT10-expressing HEK293 cell lines. Factors such as GLUT expression level, presence/absence of GLUT regulatory proteins [e.g., thioredoxin interacting protein (TXNIP)], cell type (HEK-293 cell vs. muscle), cell environment (cell culture vs. tissue), and/or membrane lipid composition could all potentially contribute to differences in inhibition by BAY-876.

In summary, the findings from this study demonstrate that in healthy mice that the muscle-specific loss of GLUT1 does not induce impairments in skeletal muscle mass, whole body metabolism, blood glucose regulation or basal skeletal muscle glucose uptake. In addition, the findings show that at low doses the chemical GLUT inhibitor, BAY-876, is selective at inhibiting muscle glucose uptake stimulated by chronic mechanical overload in the absence of changes in basal muscle glucose uptake. Last, the findings demonstrate that BAY-876 is capable of inhibiting glucose uptake via multiple facilitative glucose transporters and that its selectivity is dose dependent.

## Figures and Tables

**Table 1:** RT-qPCR primer sequence, specificity, and efficiency parameters.

Gene	GenBank Accession	Primers	Amplicon Size (bp)	Primer Efficiency	Slope	Y-Axis Intercept	R^2^ Value
SLC2A1	NM_011400	CACTGGTGTCATCAACGCCC	94	99.1%	−3.34	40.5	0.99
		AGACCAAAGCGTGGTGAGTG					
SLC2A3	NM_011401	ATGGGGACAACGAAGGTGAC	107	93.5%	−3.49	25.6	1.00
		GTCTCAGGTGCATTGATGACTC					
SLC2A6	NM_172659	CAGAGTAGCCGAGTGTCGTG	152	92.4%	−3.52	26.5	1.00
		ACCACGGATGTGTTGTCGAA					
SLC2A10	NM_130451	GCCTGACCTTCGGATATGAGC	165	90.6%	−3.57	26.9	0.99
		TGCCATAGCAGTCAATGAGGA					
HAGH	NM_001409599	CACCACTCACCACCACTGG	153	91.9%	−3.53	36.9	0.99
		ACACTGAGAGACCCCACCTG					
RPS17	NM_009092	CGGCTATGTCACGCATCTG	194	100.8%	−3.30	30.0	0.99
		TAGAGAGACTGCCAAAGTCCAGG					
SRP14	NM_009273.4	AGAGCGAGCAGTTCCTGAC	195	96.8%	−3.40	33.9	0.99
		CGGTGCTGATCTTCCTTTTC					

Sequence and size of the amplicons generated by the RT-qPCR primers for the glucose transporters GLUT1 (SLC2A1), GLUT3 (SLC2A3), GLUT6 (SLC2A6), GLUT10 (SLC2A10) and the reference genes, hydroxyacyl glutathione hydrolase (HAGH), ribosomal protein S17 (RPS17) and signal recognition particle 14 (SRP14). RT-qPCR primer efficiency was tested using cDNA isolated from mouse plantaris muscles. To assess primer efficiency for the glucose transporter genes, the mouse muscle cDNA was supplemented with plasmid cDNA encoding that specific mouse gene prior to serial dilution. RT-qPCR reactions were run using the methods described in Material and Methods.

**Table 2:** Absolute mRNA copy numbers in the glucose transporter (GLUT)-HEK293 cell lines.

Transcript Quantified	GLUT-Expressing HEK293 Cell Line						
	GLUT1	GLUT2	GLUT3	GLUT4	GLUT5	GLUT6	GLUT10
hGLUT1	462,327 ± 4566	3725 ± 326	4145 ± 501	4527 ± 768	2139 ± 180	3815 ± 29	4458 ± 822
hGLUT2	5 ± 0	84,758 ± 13,107	ND	7 ± 4	23 ± 4	540 ± 9	9 ± 4
hGLUT3	288 ± 124	403 ± 54	1,358,605 ± 33,835	1223 ± 366	205 ± 31	12,223 ± 5	2269 ± 303
hGLUT4	155 ± 76	170 ± 49	268 ± 83	288,864 ± 84,501	91 ± 15	45 ± 22	5 ± 1
hGLUT5	N/A	N/A	N/A	N/A	79,938 ± 1625	ND	ND
hGLUT6	N/A	N/A	N/A	N/A	N/A	1,532,765 ± 400,123	N/A
hGLUT10	N/A	N/A	N/A	N/A	39 ± 9	4 ± 1	40,431 ± 7235

Absolute quantification of *GLUT1*, *GLUT2*, *GLUT3*, *GLUT4*, *GLUT5*, *GLUT6* and *GLUT10* were measured by RT-qPCR in HEK293 cells stably expressing overexpressing GLUT1 or in HEK293 cells with stable knockdown of endogenous GLUT1 plus expression vector encoding the indicated human GLUT. Values are expressed in mean copies per ng cDNA ± SEM for *N* = 3 independent cultures each read in triplicate. [Legend: N/A = data not available; ND = not detected; Data from GLUT1- to GLUT4-HEK293 cell lines was taken from: [28].

## Data Availability

Not applicable.

## Supplementary Materials

The following supporting information can be downloaded at: https://www.mdpi.com/article/10.3390/biom12121734/s1, Figure S1: Body weight, body composition, skeletal muscle mass, metabolic rate, spontaneous activity, blood glucose, blood insulin, basal and insulin-stimulated glucose uptake in wild-type (WT), MCK-Cre (Cre) and GLUT1 LoxP (LoxP) mice; Figure S2: Metabolic measurements from male muscle-specific GLUT1 knockout (mG1KO) mice; Figure S3: The chemical GLUT inhibitor, BAY-876, does not impair fructose uptake via glucose transporter 5 (GLUT5); Figure S4: Testing of three commercially available glucose transporter 1 (GLUT1) antibodies for use in immunoblotting experiments.
