# Supplementary material for: Muscle-Specific Ablation of Glucose Transporter 1 (GLUT1) Does Not Impair Basal or Overload-Stimulated Skeletal Muscle Glucose Uptake
Source: Biomolecules. 2022 Nov 23;12(12):1734. doi: 10.3390/biom12121734 (PMC9776291; doi:10.3390/biom12121734)
Supplement: Supplementary file 1 [file biomolecules-12-01734-s001.zip › biomolecules-2020339-supplementary.pdf]

McMillin SL et al – *Biomolecules* – Supplemental Figure S1

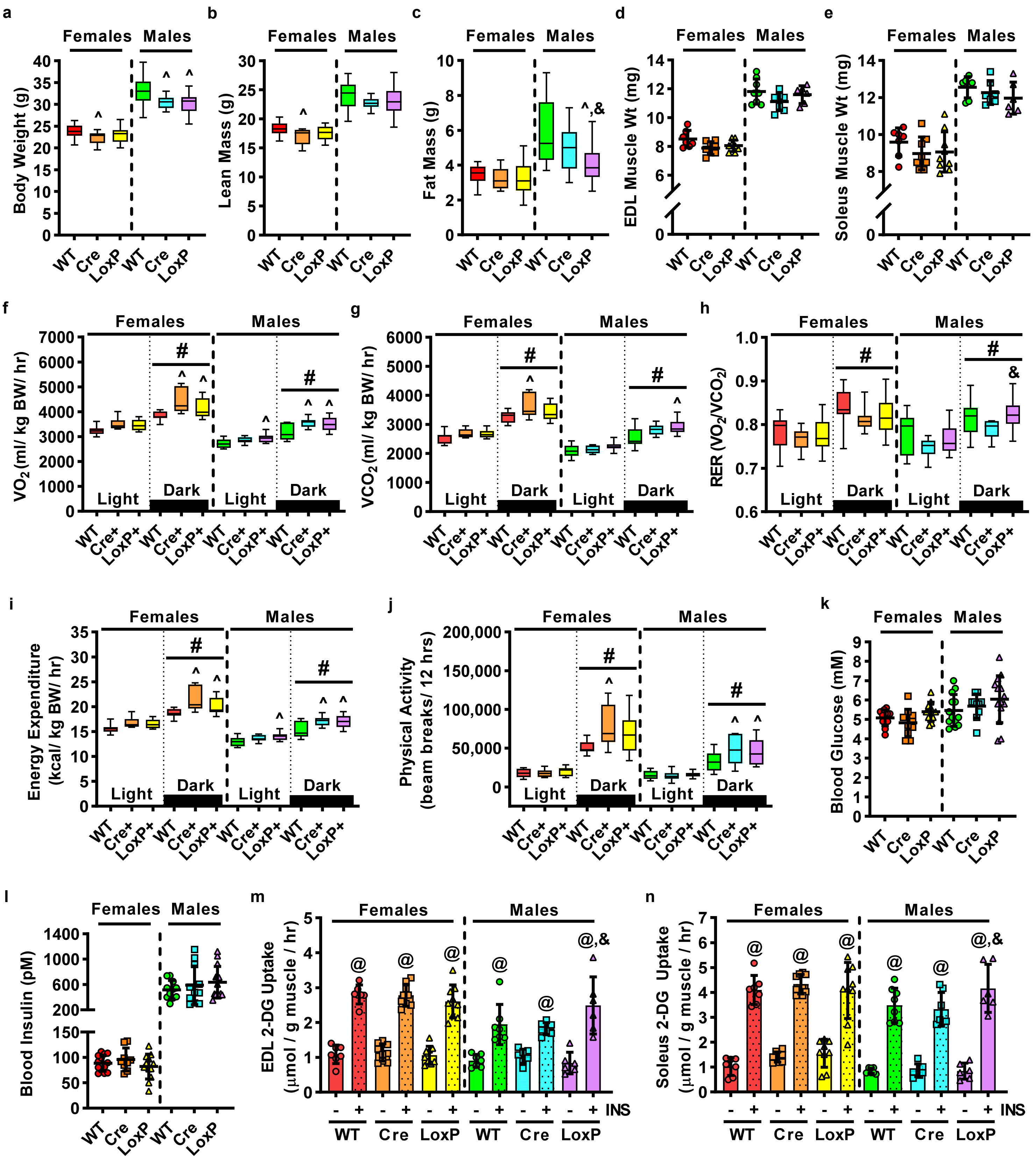

Supplemental Figure S1. Body weight, body composition, skeletal muscle mass, metabolic rate, spontaneous activity, blood glucose, blood insulin, basal and insulin-stimulated glucose uptake in wild-type (WT), MCK-Cre (Cre) and GLUT1 LoxP (LoxP) mice. All measurements were made in 11-12 wk old mice. (**a**) Mice were weighed to ± 0.1 grams. N=14-25 female mice/group & N=15-36 male mice/group. (**b-c**) Mice were placed in an EchoMRI™-700 analyzer for assessments of body composition, including: (B) lean mass; and (**c**) and fat mass. N=14-25 female mice/group & N=15-36 male mice/group. (**d-e**) Extensor digitorum longus (EDL) and soleus muscles were excised and weighed to ± 0.1 mg. N=7-9 muscles/group for females & N=6-8 muscles/group for males. (**f-j**) Mice were individually housed in Pheno/LabMaster metabolic cages at 21-22°C for indirect calorimetry measures in both the light and dark cycles. Measures included: (**f**) oxygen consumption (VO_2); (**g**) carbon dioxide production (VCO_2); (**h**) respiratory exchange ratio (RER); and (**i**) energy expenditure. (**j**) Infrared sensors detected spontaneous physical activity in the x-, y- and z-axes each hour and the average activity in a 12 hr light or dark cycle calculated. N=10-16 female mice/group & N=10-20 male mice/group. (**k-l**) Mice were fasted (12-14 hrs) overnight, and blood was collected from the tail to assess (**k**) glucose levels, and (**l**) insulin levels. N=10-13 female mice/group & N=10-14 male mice/group. (**m-n**) Mice were anesthetized with pentobarbital sodium. Muscles were excised, and [^{3}H]-2-deoxyglucose uptake assessed in the absence (-) or presence of insulin (INS; 50 mU/ml) in the (**m**) EDL, and (**n**) soleus muscles. N=7-9 muscles/group from female mice & N=6-8 muscles/group from male mice.] (**a-c** and **f-j**) Data are presented as box & whisker plots with error bars providing minimum and maximum values. (**d-e** and **k-n**) Data are presented as individual data points with the mean ± standard deviation. Statistical significance was defined as $P<0.05$, determined by one-way ANOVA with Tukey's posthoc analysis or repeated measures two-way ANOVA with Sidak's posthoc analysis and denoted by '^' vs WT, '&' vs Cre, '#' vs light cycle group effect, '@' vs no insulin (-).

McMillin SL et al – *Biomolecules* – Supplemental Figure S2

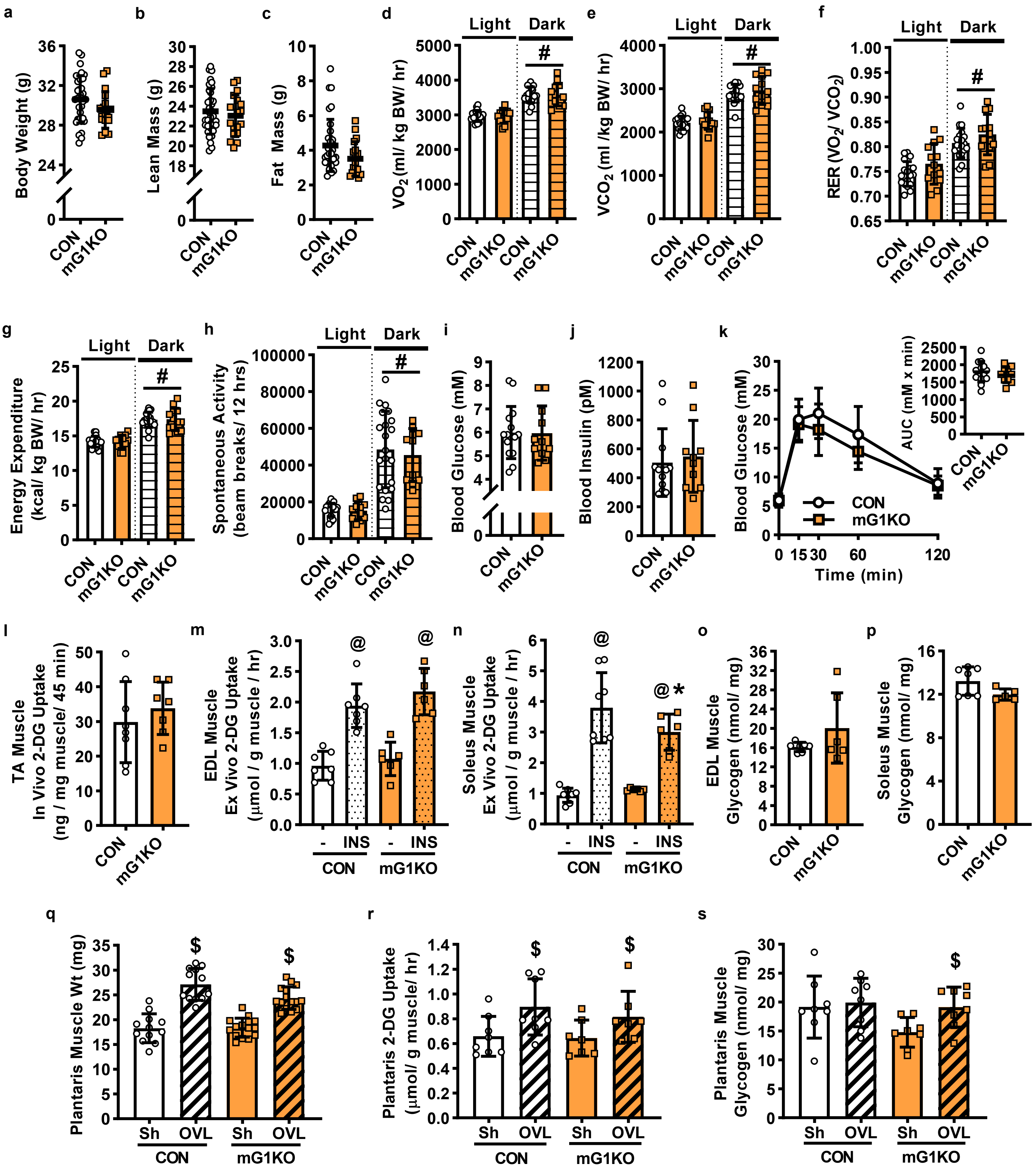

Supplemental Figure S2. Metabolic measurements from male muscle-specific GLUT1 knockout (mG1KO) mice. All measurements were made in 11-14 week old, male control (CON) and mG1KO mice. (**a**) Body weights. (**b-c**) EchoMRI™ was utilized to assess (**b**) lean mass, and (**c**) fat mass. N=18-34 mice/group. (**d-h**) Mice were individually housed in Pheno/LabMaster metabolic cages at 21-22˚C for indirect calorimetry measures in both the light and dark cycles. Measures included: (**d**) oxygen consumption (VO_2); (**e**) carbon dioxide production (VCO_2); (**f**) respiratory exchange ratio (RER); and (**g**) energy expenditure. (**h**) Infrared sensors detected spontaneous physical activity, which was calculated as the average number of beam breaks per 12 hrs. N=11-21 mice/group. (**i-k**) Mice were fasted (12-14 hrs) overnight. Blood was collected from the tail to assess (**i**) glucose, and (**j**) insulin levels. (**k**) Mice received an intraperitoneal injection of glucose (2 g/kg lean mass), and blood glucose levels measured 15, 30, 60 and 120 min later. The area under the curve was calculated (inset). N=10-14 mice/group. (**l**) Mice were fasted (12-14 hrs) overnight and anesthetized with pentobarbital sodium. Mice were injected with [^{3}H]-2-deoxyglucose (2-DG) and tibialis anterior (TA) muscles collected to assess non-stimulated in vivo muscle glucose uptake. N=7-8 muscles/group. (**m-n**) Ex vivo muscle [^{3}H]-2-deoxyglucose uptake was assessed in the absence (-) or presence of insulin (INS; 50 mU/ml) in the (**m**) extensor digitorum longus (EDL), and (**n**) soleus muscles. N=5-7 muscles/group. (**o-p**) Muscle glycogen content was assessed in the non-insulin stimulated (**o**) EDL, and (**p**) soleus muscles using a hexokinase-based assay. N=5-7 muscles/group. (**q-s**) Mice underwent unilateral synergist ablation surgery to induce plantaris muscle overload (OVL). The contralateral leg served as the control and received a sham operation. After 5 days, mice were anesthetized with pentobarbital sodium and the plantaris muscles excised. (**q**) Muscles from male mice were weighed to ±0.1 mg. N=12-15 muscles/group. (**r**) Muscles were incubated in [^{3}H]-2-deoxyglucose and glucose uptake rates calculated. N=7-8 muscles/group. (**s**) Glycogen content was assessed in muscles from male mice using a hexokinase-based assay. N=8 muscles/group. Data are presented as the mean ± standard deviation. Statistical significance was defined as $P<0.05$, assessed by t-tests (**a-c**, **i-l**, and **o-p**) and repeated measures two-way ANOVA with Sidak's posthoc analysis (**d-h**, **m-n**, and **q-s**), and denoted by '*' vs control (CON) mice, "#" vs main effect (light vs. dark cycle), '@' vs no insulin (-), or '$' vs sham-operated control muscles (Sh).

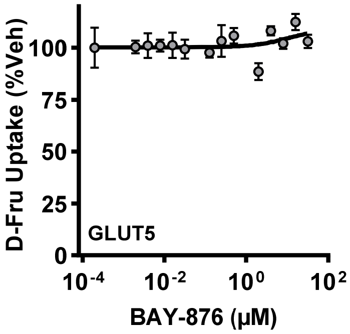

Supplemental Figure S3. The chemical GLUT inhibitor, BAY-876, does not impair fructose uptake via glucose transporter 5 (GLUT5). HEK293 cells that stably express human GLUT5 were incubated with vehicle (Veh; 0.45% DMSO) or BAY-876 for 30 min, and [^{3}H]-D-fructose uptake assessed. Data are presented as the percent of hexose uptake relative to vehicle (DMSO) treated cells ± standard error, and the IC_{50} values determined using least squares non-linear regression analyses. N=3 independent experiments, N=3 replicates per experiment.

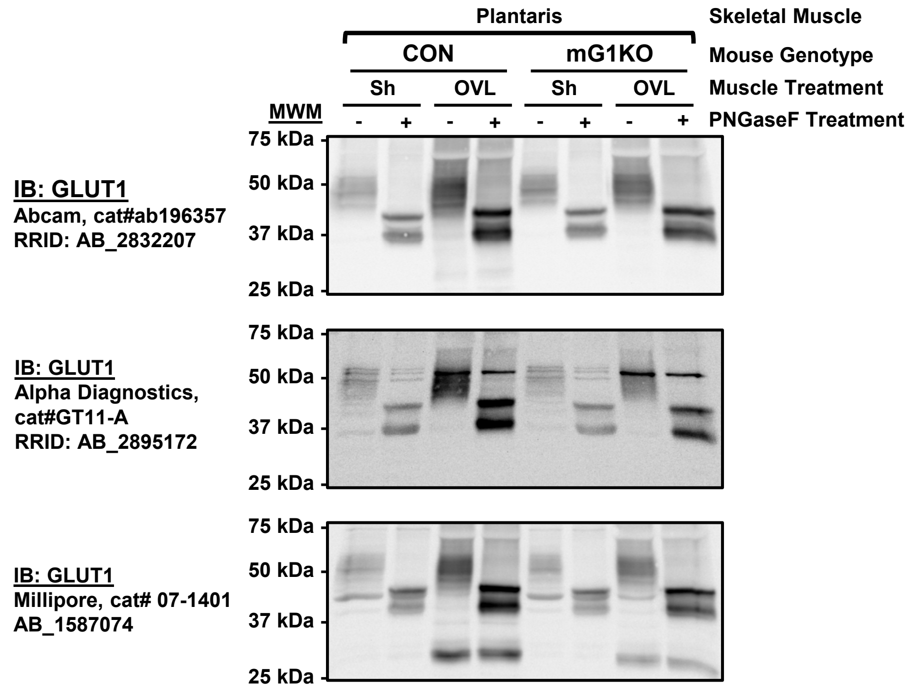

Supplemental Figure S4. Testing of Three Commercially Available Glucose Transporter 1 (GLUT1) Antibodies for Use in Immunoblotting Experiments. Female, control (CON) and muscle-specific GLUT1 knockout (mG1KO) mice underwent unilateral synergist muscle ablation surgery to induce plantaris muscle overload (OVL). The contralateral leg served as the control and received a sham operation (Sh). After 5 days, plantaris muscles were excised and processed for immunoblot analysis. Lysates were incubated in the presence or absence of the deglycosylation enzyme, PNGase F, to remove glycosylated moieties from GLUT1 proteins. Representative images.
